# Predictors of Major Incident Fracture During Adulthood: Evidence from the Geelong Osteoporosis Study

**DOI:** 10.1007/s00223-026-01638-z

**Published:** 2026-10-29

**Authors:** Aminu Suleiman, Mojtaba Lotfaliany, Lana J. Williams, Kara B. Anderson, Amanda L. Stuart, Kara L. Holloway-Kew, Julie A. Pasco

**Affiliations:** 1 School of Medicine, Deakin Institute for Mental and Physical Health and Clinical Translation (Deakin IMPACT), School of Medicine, Deakin University, Geelong, VIC 3220 Australia; 2 Barwon Health, Geelong, VIC 3220 Australia; 3 Department of Epidemiology and Preventive Medicine, Monash University, Melbourne, VIC 3220 Australia

**Keywords:** Time-dependent Cox regression, Incident fractures, Musculoskeletal ageing, Healthy ageing

## Abstract

As people age, preventing fractures becomes crucial for preserving independence and overall quality of life. This study aimed to identify factors associated with reduced hazard of major incident fractures during adulthood. Serial data from 1394 females and 1404 males (ages 20-96y) participating in the Geelong Osteoporosis Study were analysed. Fractures (hip, spine, proximal humerus, distal radius, pelvis) were identified through radiological reports. Sociodemographic and lifestyle factors, Anthropometry, dual-energy X-ray absorptiometry, comorbidities, medication use and biochemical markers were collected across the visits. Predictors of major incident fractures were examined using time-dependent Cox proportional hazards models with age as the primary time scale. Participants were followed from baseline until first fracture, death, loss to follow-up or 31/12/2020. Stepwise forward selection identified relevant predictors, and missing data were imputed using predictive mean matching. Over a median follow-up of 16.3y (IQR:10.5–23.5), fractures occurred in 430 participants (15.4%). Males had a lower fracture hazard than females (HR 0.71, 95% CI 0.52–0.97). Factors associated with a reduced hazard of fracture included never-smoking (0.70, 0.50–0.97), past smokers (0.67, 0.46–0.99), higher hip and spine T-scores (0.82, 0.72–0.93; 0.82, 0.76–0.89), lower plasma glucose (0.60, 0.42–0.87), lower HDL cholesterol (0.69, 0.52–0.90), absence of falls (0.74, 0.59–0.91), no muscle weakness (0.48, 0.23–0.98) and non-use of oral glucocorticoids (0.48, 0.31–0.75). Preserving bone mass, undertaking falls prevention strategies, avoiding smoking, monitoring oral glucocorticoid use and optimising metabolic health could be possible strategies for reducing fracture risk during ageing. The independent contribution of HDL might provide new perspectives into fracture risk assessment.

## Introduction

Fractures are a serious public health concern that poses a significant burden. The substantial implications of fractures include pain, disability, mortality and healthcare expenses [1, 2]. The burden of fractures has continued to rise, driven by increases in life expectancy and societal ageing, particularly in developed nations [3]. In Australia, there was one fracture reported every 2.7 min in 2023, and this burden is projected to rise sharply to one fracture every minute by 2033 [4]. Over the next decade, the cumulative number of fractures, including both new and follow-up cases, is expected to exceed 2.1 million [4], highlighting the substantial and growing impact of fractures on the Australian population. Therefore, determining factors that increase the risk of these fractures is essential for developing preventive measures and improving quality of life for those who are vulnerable [1].

Bone health can be influenced by a complex interaction of genetic, clinical, lifestyle and environmental factors [5, 6]. Previous studies have established that a lower bone mineral density (BMD) is associated with a higher risk of fractures, especially in the older population [7, 8]. Preserving muscle mass and regular physical activity are also important to preserve bone health and help prevent falls, which are a primary cause of fractures [9, 10]. Furthermore, comorbid conditions such as cardiovascular diseases and diabetes mellitus have previously been associated with bone health and fracture risk [11].

Despite the known risk factors for fracture, more research is needed to determine and quantify the factors that contribute to adult fracture-free survival. This entails investigating new predictors, in addition to standard risk factor analysis. Insights into how these variables interact over time to affect fracture risk have been made possible by recent developments in time-dependent covariate modelling and longitudinal data analysis [12]. These highlight the importance of identifying time-plausible variables that can help individuals avoid fractures as they age. Comprehending these variables is critical not only for enhancing individual health care outcomes but for reducing the social and economic burden of fractures. In this study, comprehensive longitudinal data and advanced statistical techniques are used to identify factors associated with major incident fractures during adulthood.

## Methods

### Participants

Data for this study were collected from participants enrolled in the Geelong Osteoporosis Study (GOS): 1494 females were recruited between 1993 and 1997, and 1539 males between 2001 and 2006. Participant ages ranged from 20 to 96 years at baseline. GOS is a population-based cohort study carried out in southeastern Australia. Participants were selected at random from the Australian electoral roll of the Barwon Statistical Division. An in-depth description of the GOS study has been published elsewhere [13]. Informed written consent was provided by all participants. The study was approved by the Barwon Health Human Research Ethics Committee (approval numbers 92/01 and 00/56). For this study, women contributed data from baseline, 10-year and 15-year follow-ups, and men from baseline, 5-year and 15-year follow-ups.

**Fig. 1:**
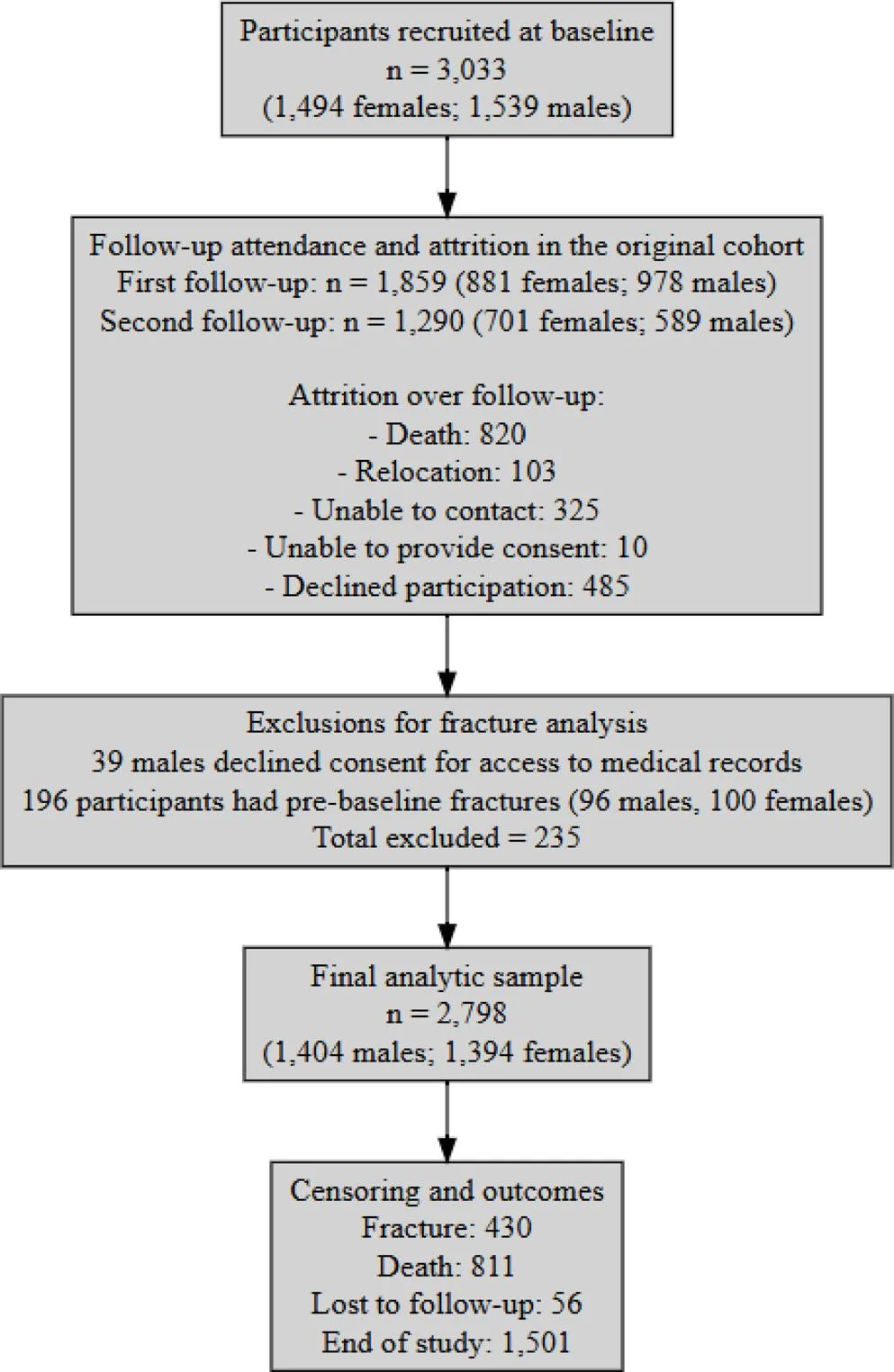
Participant flow chart detailing sex-specific recruitment, exclusions, follow-up waves, and final censoring outcomes

Among the 3033 participants (1494 females and 1539 males) recruited at baseline, 1859 individuals (881 females and 978 males; median age 58.6 years) attended the first follow-up assessment, decreasing to 1290 participants (701 females and 589 males; median age 62.3 years) at the second follow-up assessment. Attrition across the follow-up period was primarily attributable to death (*n* = 820), relocation outside the study region (*n* = 103), inability to be contacted (*n* = 325), inability to provide consent (*n* = 10) or declining further participation (*n* = 485). For the present fracture analysis, 39 opted out of consent to access medical records, and an additional 96 males and 100 females with pre-baseline fractures (hip, clinical spine, proximal humerus, distal radius or pelvis) were excluded. This resulted in a final analytic sample of 2798 participants (1404 males and 1394 females). The analysis examined the occurrence of a first major incident fracture from baseline until the end of the study period (31 December 2020). During follow-up, final censoring and event outcomes comprised 430 incident fractures, 811 deaths and 56 participants lost to follow-up, while 1,501 participants remained fracture-free and under observation until the end of the study period (Fig. 1).

## Measures

### Outcome Variables

#### Fracture

Radiological reports from radiology centres in the study region were reviewed to identify incident fractures that occurred during follow-up, as previously described [14]. Fractures at major sites (hip, clinical spine, proximal humerus, pelvis and distal radius) were included irrespective of trauma. These fracture sites align with sites used in the standard definition of major osteoporotic fractures, except for the pelvis, which recent literature recommends including due to its similar clinical and prognostic characteristics [15]. The adult age range in this cohort was 20 to 96 years; therefore, the conventional definition of major osteoporotic fractures, which is typically applied to older adults, was not appropriate. To avoid misclassification across this wide age range, fractures were classified solely by anatomical site rather than presumed mechanism, and the neutral term major incident fractures was used throughout the manuscript. Informed written consent was obtained to access participants’ medical records.

#### Death

Mortality data were obtained from the National Death Index through data linkage with the Australian Institute of Health and Welfare (AIHW).

### Exposure Variables

#### Demographic Variables

The Index of Relative Socioeconomic Disadvantage (IRSD) was used as the measure of socioeconomic status (SES). Participants’ residential addresses were matched to Australian Bureau of Statistics census data to assign an IRSD score. These scores were grouped into quintiles, with quintile 1 representing the most disadvantaged areas. In this analysis, SES was regrouped into three categories to improve interpretability: quintiles 1 and 2 were categorised as low SES, quintile 3 as medium SES and quintiles 4 and 5 as high SES.

#### Anthropometric and Clinical Measures

Weight was measured in kilograms (kg) using an electronic weighing scale and height in centimetres (cm) using a Harpenden stadiometer at each assessment phase. Body mass index (BMI) was calculated as weight divided by height in metres squared (kg/m^2^). Waist circumference was measured in centimetres (cm) using a standard anthropometric tape in each visit. For waist circumference and height, values were reverse-coded to explore the influence of lower values.

Measures of bone mineral density (BMD) at the hip and lumbar spine, as well as appendicular lean mass (ALM), were assessed using dual-energy X-ray absorptiometry (DXA; Lunar, Madison, WI, USA) at each assessment phase. At baseline and the 10-year follow-up, BMD in females was measured using a DPX-L densitometer, with a Prodigy Pro used at the 15-year follow-up. For males, the DPX-L densitometer was used at baseline until the device was replaced by the Prodigy Pro, which was used thereafter. Cross-calibration between the densitometers was performed to ensure measurement consistency across devices and time points [13]. This process involved phantom scanning and regression-based adjustment, thereby minimising systematic bias and preserving longitudinal comparability. BMD values were converted to T-scores using Australian reference data for young adults [16, 17]. ALM was calculated in kilograms (kg) as the sum of bilateral lean mass in the arms and legs. Height-adjusted ALM (ALM/height²) was then derived by dividing ALM by height in metres squared (m^2^). All measurements were performed by trained personnel.

#### Lifestyle Factors

At all visits, self-reported lifestyle variables included alcohol consumption, tobacco smoking, physical activity status and dietary calcium intake. Alcohol consumption and physical activity were treated as binary variables, while smoking was categorised as past, current or never. Current smokers were used as the reference group. Alcohol intake was categorised as low (never, less than once per week or once to twice per week) or high (several times per week or daily). Physical activity was reported on a seven-point scale and re-classified as active (very active or active) or sedentary (sedentary, limited, inactive, chair-bound, bedridden or bedfast). Dietary calcium intake calculated in grams per day (g/day) was obtained from a validated questionnaire at baseline [18], and subsequently by the Dietary Questionnaire for Epidemiological Studies (DQES v3.2), designed by Cancer Council Victoria. Dietary calcium was initially calculated in milligrams per day and converted to grams per day (g/day) to improve interpretability.

#### Biochemical Markers

Blood samples were collected following an overnight fast at baseline and the 10-year follow-up for females, and at baseline and the 15-year follow-up for males. Aliquots of serum and plasma were stored at − 80 °C until batch analysis. Fasting plasma glucose and lipid concentrations, including triglycerides (TG), low-density lipoprotein (LDL), high-density lipoprotein (HDL) and total cholesterol, were measured. Serum PINP (procollagen type I N-terminal propeptide) and β-CTX-I (β-isomerized C-terminal telopeptide of type I collagen) were measured using Roche Diagnostics immunoassays on the Roche Modular Analytics E170 analyser (Roche Diagnostics, Germany). For the statistical models, these biomarkers were reverse-coded to examine protective associations of lower concentrations. Plasma glucose was converted to a categorical variable, with values < 7 mmol/L classified as low and values ≥ 7 mmol/L as high plasma glucose.

#### Medications

Medications such as oral glucocorticoids use, thyroid hormone replacement therapy, gonadal hormone replacement therapy and agents affecting calcium metabolism were self-reported at each visit. In the analyses, medication use was referenced against non-use to evaluate the effect of medication exposure.

#### Comorbid Conditions

During each follow-up visit, comorbid conditions, including hypertension, cardiac arrhythmias, stroke, emphysema, asthma, bronchitis, falls over the preceding 12 months, and prior fractures were self-reported by participants. Cancer cases were confirmed through data linkage with the Victorian Cancer Registry. A combination of systolic blood pressure of ≥ 140 mmHg or a diastolic blood pressure of ≥ 90 mmHg or self-reported hypertension in conjunction with current medication use (beta-adrenergic blocking drugs, diuretics and antihypertensives) was used to define hypertension. These comorbid conditions were referenced against the presence group in the statistical models.

### Statistical Analysis

Participant characteristics are presented as count (n) and percentage (%) for categorical variables, mean and standard deviation for normally distributed continuous variables, or median and interquartile range (IQR) for non-parametric variables. Comparison of baseline characteristics was performed between fracture and non-fracture groups using the T-test for continuous variables, the Mann-Whitney *U* test for non-parametric continuous variables, and the Chi-square test for categorical variables.

A time-dependent Cox proportional hazards model was used to identify predictors of major incident fracture, with age as the primary time scale. To prevent delayed entry (left truncation) bias, models were constructed using a counting process framework (Surv(time, time2, event)) in R, where each participant’s timeline was explicitly bounded by their exact age at the start of an observation interval and their exact age at interval exit. Participants were followed from baseline until first fracture, death, loss to follow-up or the end of the study period (31 December 2020). For time-varying analyses, follow-up was split into intervals defined by consecutive attended study visits. Each interval began at an attended visit and ended at the next attended visit or at fracture, death, loss to follow-up or end of study, whichever occurred first. Covariate values were replaced by updated measurements at the start of each attended follow-up visit and carried forward until the end of the corresponding observation interval, ensuring that each interval reflected the most recent information available. Participants remained under follow-up even if an intermediate clinic visit was missed and continued to contribute person-time until fracture, death, loss to follow-up or the end of the study period. Participants who remained in the study without fracture, death or loss to follow-up contributed person-time until study completion. When a participant attended a visit but had missing covariate values, these were imputed using predictive mean matching; imputation filled missing values within existing visits but did not generate additional follow-up intervals. Total person-years represent the sum of interval-specific follow-up times across all participants from interval entry to interval exit, and median follow-up was derived from the same time-to-event data, reflecting the duration participants remained under observation before fracture or censoring.

All candidate predictors were entered into the same initial model, and stepwise regression based on the lowest Akaike Information Criterion (AIC) was applied to this full set to identify the most relevant predictors of fracture. Missing covariate data were imputed using predictive mean matching (PMM) in the *mice* package in R. A single imputation (m = 1) was generated, with the imputation model including all covariates across all available waves; survival time and event status were fully observed and therefore not imputed. Sensitivity analyses comparing imputed and complete-case models yielded consistent estimates. To address potential multicollinearity, correlations among predictors were examined and highly correlated variables were not included simultaneously; variance inflation factors (VIFs) were assessed in the final model to confirm that no substantial collinearity remained. The proportional hazards assumption was evaluated using Schoenfeld residuals for all Cox models.

To assess whether associations differed in older adults, we conducted an age-stratified sensitivity analysis comparing participants aged 20–64 years with those aged ≥ 65 years. The ≥ 65-year threshold reflects the age group in which fractures at major osteoporotic sites predominantly indicate skeletal fragility. All time-dependent Cox models were re-estimated within each age stratum using the same covariate structure as the primary analysis. Additionally, stepwise AIC selection was applied within each age stratum. This allowed identification of predictors that contributed most strongly to fracture hazard in each age group. This approach enabled evaluation of whether predictors demonstrated different associations with fracture hazard between mid-life adults and older adults. R version 4.3.1 was used to perform analyses.

## Results

During follow-up, the cohort accumulated 47,956 person-years of observation. The median follow-up duration of the study was 16.3 years (IQR: 10.5–23.5 years). In total, 430 incident fractures were identified, with females accounting for 284 (66.0%) and males for 146 (34.0%). For both sexes, clinical spine fractures were most prevalent (*n* = 200, 46.5%), followed by distal radius (*n* = 102, 23.7%), hip (*n* = 79, 18.3%), pelvis (*n* = 32, 7.4%) and proximal humerus fractures (*n* = 17, 4.0%).

Table 1 presents the baseline characteristics of the study population. Compared to those who remained fracture-free, participants with fractures were older and more likely to be female. They also had lower physical activity, lower dietary calcium intake, lower height, lower ALM/h^2^ and lower bone mineral density at the spine and hip. Higher glucose, total cholesterol, LDL and PINP levels were observed among those with fractures. Comorbidities such as osteoarthritis, hypercholesterolemia, hypertension, cancer, and emphysema were more frequent in the fracture group. The use of oral glucocorticoids and gonadal hormones was also more frequent among those with fractures. No significant differences were observed for BMI, waist circumference, smoking, alcohol intake, falls, serum β-CTX-I, use of thyroid hormones or use of agents affecting calcium metabolism.

**Table 1:** Baseline characteristics of the participants stratified by incidence of fragility fractures. Data are presented as *mean (SD)* for normally distributed variables and *median (IQR)* for skewed variables. Categorical variables are shown as *n (%)*

Variables	All (*n* = 2798)	Fracture-free (*n* = 2368)	Fracture (*n* = 430)	*p* value
Demographic and lifestyle factors				
Age (y)	54.2 (37.8, 71.7)	51.1 (36.0, 69.4)	69.6 (57.1, 77.9)	< 0.001
Male sex	1,404 (50.2%)	1,258 (53.1%)	146 (34.0%)	< 0.001
Low SES (ref)	1,101 (39.3%)	933 (39.4%)	168 (39.1%)	0.063
Medium SES	561 (20.1%)	458 (19.3%)	103 (24.0%)	
High SES	1,136 (40.6%)	977 (41.3%)	159 (37.0%)	
Past smoker	937 (33.5%)	788 (33.3%)	149 (34.7%)	0.065
Current smoker	444 (15.9%)	392 (16.6%)	52 (12.1%)	
High alcohol consumption	887 (31.7%)	747 (31.5%)	140 (32.6%)	0.678
Physically active	2000 (71.5%)	1740 (73.5%)	260 (60.5%)	< 0.001
Dietary calcium intake (g/day)	0.8 (0.5, 1.0)	0.8 (0.5, 1.0)	0.7 (0.5, 1.0)	0.039
Anthropometric and body composition measurements				
BMI (kg/m^2^)	26.2 (23.5, 29.3)	26.2 (23.5, 29.3)	26.0 (23.6, 29.3)	0.542
Height (cm)	167.6 (10.0)	168.3 (10.0)	163.6 (9.4)	< 0.001
Weight (kg)	75.5 (16.2)	76.2 (16.2)	71.7 (15.5)	< 0.001
ALM/height^2^ (kg/m^2^)	7.5 (6.5, 8.6)	7.6 (6.6, 8.7)	6.8 (6.1, 7.8)	< 0.001
Waist circumference (cm)	91.0 (13.8)	91.1 (13.7)	90.7 (14.1)	0.559
Hip T-score	− 0.8 (1.2)	− 0.7 (1.2)	− 1.5 (1.2)	< 0.001
Spine T-score	− 0.3 (1.5)	− 0.1 (1.4)	− 1.0 (1.6)	< 0.001
Biochemical markers				
Total cholesterol (mmol/L)	5.3 (1.1)	5.3 (1.1)	5.5 (1.2)	< 0.001
HDL (mmol/L)	1.3 (0.3)	1.3 (0.3)	1.3 (0.4)	0.091
LDL (mmol/L)	3.0 (0.9)	3.0 (0.9)	3.1 (0.9)	0.177
Triglycerides (mmol/L)	1.5 (0.9)	1.5 (0.9)	1.4 (0.7)	0.547
β-CTX-I (ng/L)	337.0 (227.2, 477.9)	331.1 (226.5, 471.7)	372.8 (236.2, 505.5)	0.061
PINP (µg/L)	37.0 (27.0, 51.0)	37.0 (27.0, 51.0)	39.5 (28.0, 51.8)	0.019
Plasma glucose > = 7 mmol/L	112 (4.0%)	85 (3.6%)	27 (6.3%)	0.009
Medical conditions				
Osteoarthritis	286 (10.2%)	214 (9.0%)	72 (16.7%)	< 0.001
Falls	604 (21.6%)	500 (21.1%)	104 (24.2%)	0.154
Hypertension	1119 (40.0%)	905 (38.2%)	214 (49.8%)	< 0.001
Transient weakness	40 (1.4%)	34 (1.4%)	6 (1.4%)	0.948
Stroke	87 (3.1%)	70 (3.0%)	17 (4.0%)	0.273
Cardiac arrhythmias	70 (2.5%)	54 (2.3%)	16 (3.7%)	0.078
Asthma	377 (13.5%)	309 (13.0%)	68 (15.8%)	0.122
Emphysema	50 (1.8%)	35 (1.5%)	15 (3.5%)	0.004
Bronchitis	78 (2.8%)	64 (2.7%)	14 (3.3%)	0.522
Cancer	299 (10.7%)	234 (9.9%)	65 (15.1%)	0.001
Muscle weakness	36 (1.3%)	28 (1.2%)	8 (1.9%)	0.251
Medication use				
Oral glucocorticoids	51 (1.8%)	30 (1.3%)	21 (4.9%)	< 0.001
Gonadal hormone	158 (5.6%)	122 (5.2%)	36 (8.4%)	0.008
Thyroid hormone	43 (1.5%)	34 (1.4%)	9 (2.1%)	0.308
Agent affecting calcium	32 (1.1%)	21 (0.9%)	11 (2.6%)	0.003

*P* values compare participants with and without fractures using χ^2^ tests for categorical variables and t tests or Mann–Whitney U tests for continuous variables, as appropriate

Medications included in the “agents affecting calcium” category: Vitamin D supplements, bisphosphonates (including risedronate, alendronate, etidronate and denosumab), selective estrogen reuptake inhibitors, strontium ranelate

ALM/h^2^, appendicular lean mass divided by height squared; BMI, body mass index; BMD, bone mineral density; (β-CTX-I), β-isomerized C terminal telopeptide of type I collagen; HDL, high density lipoprotein; hsCRP, high sensitivity C reactive protein; IQR, interquartile range; LDL, low density lipoprotein; PINP, procollagen type I N terminal propeptide; SES, socioeconomic status

### Multivariable Model

In a time-dependent Cox model including all covariates, several meaningful associations with the hazard of major incident fractures were identified. Males experienced a 29% reduced hazard of fracture compared with females. Both never and past smokers demonstrated reduced hazard compared to current smokers, with 30% and 33% reduced hazards, respectively. Lower HDL cholesterol was associated with a 31% reduction in fracture hazard, with each 1-mmol/L decrease corresponding to a hazard ratio of 0.69. Participants with no history of falls had a 26% lower hazard. Higher femoral neck and spine T scores were associated with a reduced hazard of fracture, with each one-SD increase corresponding to an 18% lower hazard (HR 0.82 for both sites). Low plasma glucose was associated with a 40% lower hazard (HR 0.60). Both non-use of oral glucocorticoids and absence of muscle weakness were associated with a 52% reduction in fracture hazard. Absence of asthma was associated with a 23% lower fracture hazard, and lower waist circumference with a 51% lower hazard (HR 0.49), although neither association reached conventional statistical significance (Table 2).

**Table 2:** Time-dependent multivariable Cox regression model for predictors of major incident fractures

Predictors	HR’s	95%CI	*p*
Male sex	0.71	0.52–0.97	**0.034**
Medium SES	1.16	0.90–1.49	0.256
High SES	1.02	0.82–1.28	0.849
Never smoked	0.70	0.50–0.97	**0.033**
Past smoker	0.67	0.46–0.99	**0.044**
Low alcohol consumption	1.00	0.79–1.25	0.968
Physically active	0.86	0.69–1.07	0.188
Dietary calcium intake (g/day)	0.85	0.65–1.10	0.215
Low waist circumference (m)	0.49	0.20–1.20	0.116
Low height (m)	0.48	0.11–2.18	0.343
Hip T-score	0.82	0.72–0.93	**0.002**
Spine T-score	0.82	0.76–0.89	**< 0.001**
Low serum HDL (mmol/L)	0.69	0.52–0.90	**0.007**
Low plasma glucose (< 7mmol/L)	0.60	0.42–0.87	**0.007**
Non-fallers	0.74	0.59–0.91	**0.005**
No osteoarthritis	0.97	0.76–1.23	0.805
No hypertension	1.11	0.90–1.37	0.327
No transient weakness	0.83	0.34–2.06	0.692
No stroke	0.97	0.63–1.48	0.873
No cardiac arrhythmias	0.92	0.59–1.43	0.716
No asthma	0.77	0.59–1.00	**0.050**
No emphysema	0.72	0.43–1.19	0.194
No bronchitis	1.34	0.78–2.32	0.290
No cancer	1.08	0.85–1.39	0.525
No muscle weakness	0.48	0.23–0.98	**0.044**
Non-use of oral glucocorticoids	0.48	0.31–0.75	**0.001**
Non-use of gonadal hormone	0.91	0.58–1.41	0.667
Non-use of thyroid hormone	1.15	0.64–2.06	0.637
Non-use of agent affecting calcium	0.85	0.51–1.42	0.539

Hazard ratios (HRs) and 95% confidence intervals (95% CI) were estimated using time-dependent Cox regression. Reference categories were: smoking = current smoker; socioeconomic status = low SES; alcohol intake = high; physical activity = inactive; plasma glucose > 7 mmol/L; and falls, osteoarthritis, hypertension, stroke, cardiac arrhythmia, asthma, emphysema, bronchitis, cancer and muscle weakness. Medication reference groups were use of statins, oral glucocorticoids, gonadal hormones, thyroid hormones, and calcium-affecting agents. Selected continuous variables (dietary calcium intake, height, HDL cholesterol, and plasma glucose) were inverted to examine the influence of lower concentrations on fracture risk. Values in bold indicate statistical significance (*p* < 0.05)

BMI, body mass index; HDL, high-density lipoprotein; SES, socioeconomic status; HR, hazard ratio; CI, confidence interval

In the stepwise selected model, the most significant predictors remained consistent with those identified in the full model (Table 3). Males again demonstrated a 22% reduced hazard of fractures. Higher hip and spine T scores continued to show a robust association with lower hazard of fracture, with each one-SD increase corresponding to an 18% lower hazard (HR 0.82 for both sites). Non-use of oral glucocorticoids remained a strong predictor, associated with a 51% lower hazard. Being a non-faller was associated with a 26% reduction in hazard (HR 0.74), and low plasma glucose with a 40% reduction (HR 0.60). Lower HDL cholesterol again demonstrated a 32% lower hazard, with each 1-mmol/L decrease corresponding to a hazard ratio of 0.68. Past and never smokers continued to exhibit 32–33% lower hazard. Being physically active, having a lower waist circumference, no asthma history and no muscle weakness were associated with lower fracture hazard, with trends of 15%, 50%, 22% and 47%, respectively; however, these associations did not reach statistical significance. They were retained in the stepwise Cox regression model, likely reflecting their contribution to overall fracture prediction.

**Table 3:** Predictors selected by stepwise Cox regression using forward selection for protective factors of fractures

Predictors	HR’s	95%CI	*p*
Male sex	0.78	0.61–0.99	**0.044**
Never smoked	0.68	0.49–0.95	**0.023**
Past smoker	0.67	0.46–0.99	**0.043**
Physically active	0.85	0.69–1.06	0.149
Low waist circumference (m)	0.50	0.21–1.20	0.122
Hip T-score	0.82	0.73–0.93	**0.002**
Spine T-score	0.82	0.76–0.89	**< 0.001**
Low serum HDL (mmol/L)	0.68	0.52–0.88	**0.004**
Low plasma glucose (< 7mmol/L)	0.60	0.42–0.86	**0.006**
Non-faller	0.74	0.60–0.92	**0.006**
No asthma	0.78	0.60–1.02	0.070
No muscle weakness	0.53	0.26–1.07	0.076
Non-use of oral glucocorticoids	0.49	0.32–0.76	**0.001**

Hazard ratios (HRs) and 95% confidence intervals (95% CI) were estimated using time-dependent Cox regression. Reference categories were: smoking = current smoker; socioeconomic status = low SES; alcohol intake = high; physical activity = inactive; plasma glucose > 7 mmol/L; and falls, osteoarthritis, hypertension, stroke, cardiac arrhythmia, asthma, emphysema, bronchitis, cancer and muscle weakness. Medication reference groups were use of statins, oral glucocorticoids, gonadal hormones, thyroid hormones, and calcium-affecting agents. Selected continuous variables (dietary calcium intake, height, HDL cholesterol, and plasma glucose) were inverted to examine the influence of lower concentrations on fracture risk. Values in bold indicate statistical significance (*p* < 0.05)

HDL, high-density lipoprotein; HR, hazard ratio; CI, confidence interval

Age-stratified sensitivity analyses demonstrated clear differences in predictors of major incident fractures between mid-life adults (20–64 years) and older adults (≥ 65 years). Among adults aged 20–64 years, spine T-score (HR 0.79, 95% CI 0.66–0.95, *p* = 0.011), low HDL (HR 0.57, 95% CI 0.33–0.97, *p* = 0.038), low plasma glucose (HR 0.34, 95% CI 0.15–0.75, *p* = 0.008), asthma (HR 0.44, 95% CI 0.28–0.68, *p* < 0.001), muscle weakness (HR 0.26, 95% CI 0.08–0.88, *p* = 0.030) and non-use of oral glucocorticoids (HR 0.31, 95% CI 0.12–0.76, *p* = 0.011) were independently associated with lower fracture hazard. Hip T-score showed a borderline association (HR 0.80, 95% CI 0.62–1.02, *p* = 0.069). In contrast, among adults aged ≥ 65 years, male sex (HR 0.67, 95% CI 0.46–0.98, *p* = 0.039), hip T-score (HR 0.83, 95% CI 0.72–0.96, *p* = 0.012), spine T-score (HR 0.84, 95% CI 0.77–0.92, *p* < 0.001), non-faller status (HR 0.74, 95% CI 0.58–0.95, *p* = 0.017) and non-use of oral glucocorticoids (HR 0.51, 95% CI 0.31–0.86, *p* = 0.011) were the principal predictors. Low HDL showed a borderline association (HR 0.73, 95% CI 0.54–1.00, *p* = 0.052). Several predictors that were significant among participants aged 20–64 years, particularly asthma, low plasma glucose and muscle weakness, were not independently associated with fracture hazard in older adults, likely reflecting overlap with frailty-related pathways in later life. Conversely, hip T-score and fall history showed stronger associations in the ≥ 65 group, consistent with the predominance of fragility fractures in older adults. Overall, the pattern of predictors differed across age strata, indicating that metabolic, respiratory and neuromuscular factors were more relevant in participants aged 20–64 years, whereas skeletal fragility and fall-related factors were more influential in older adults (Table 4).

**Table 4:** Time-dependent multivariable Cox regression model for predictors of major incident fractures, stratified by age group

Predictors	20–64 years			Above 65 years		
	HR’s	95%CI	*p*	HR’s	95%CI	*p*
Male sex	0.79	0.42–1.48	0.464	0.67	0.46–0.98	**0.039**
Medium SES	1.44	0.85–2.41	0.172	1.05	0.78–1.42	0.729
High SES	1.16	0.74–1.83	0.517	0.96	0.74–1.25	0.787
Never smoked	0.68	0.42–1.12	0.133	0.67	0.42–1.07	0.091
Past smoker	0.73	0.38–1.39	0.335	0.64	0.38–1.07	0.088
Low alcohol consumption	0.98	0.63–1.52	0.919	0.98	0.75–1.27	0.872
Physically active	0.96	0.59–1.55	0.858	0.83	0.64–1.07	0.144
Dietary calcium intake (g/day)	0.87	0.55–1.37	0.555	0.83	0.60–1.14	0.251
Low waist circumference (m)	0.42	0.08–2.16	0.296	0.52	0.18–1.57	0.249
Low height (m)	0.42	0.02–9.46	0.585	0.43	0.07–2.52	0.350
Hip T-score	0.80	0.62–1.02	0.069	0.83	0.72–0.96	**0.012**
Spine T-score	0.79	0.65–0.96	**0.016**	0.84	0.77–0.92	**< 0.001**
Low serum HDL (mmol/L)	0.57	0.33–0.97	**0.038**	0.73	0.54–1.00	0.052
Low plasma glucose (< 7mmol/L)	0.34	0.15–0.75	**0.008**	0.68	0.44–1.03	0.072
Non-fallers	0.74	0.48–1.16	0.192	0.74	0.58–0.95	**0.017**
No osteoarthritis	0.86	0.48–1.52	0.596	1.01	0.78–1.32	0.916
No hypertension	1.42	0.90–2.24	0.130	1.10	0.86–1.40	0.464
No transient weakness	1.60	0.20–12.59	0.654	0.68	0.24–1.87	0.450
No stroke	0.43	0.10–1.83	0.251	1.01	0.65–1.58	0.955
No cardiac arrhythmias	1.69	0.40–7.18	0.480	0.84	0.52–1.34	0.462
No asthma	0.44	0.28–0.68	**< 0.001**	1.03	0.73–1.47	0.857
No emphysema	0.57	0.16–2.11	0.402	0.73	0.42–1.29	0.282
No bronchitis	1.04	0.45–2.40	0.920	2.01	0.89–4.56	0.095
No cancer	0.90	0.48–1.69	0.744	1.12	0.85–1.46	0.419
No muscle weakness	0.26	0.08–0.88	**0.030**	0.53	0.22–1.27	0.155
Non-use of oral glucocorticoids	0.31	0.12–0.76	**0.011**	0.51	0.31–0.86	**0.011**
Non-use of gonadal hormone	1.21	0.59–2.49	0.610	0.81	0.44–1.46	0.474
Non-use of thyroid hormone	1.18	0.28–4.95	0.822	1.15	0.61–2.19	0.662
Non-use of agent affecting calcium	0.50	0.11–2.16	0.351	0.88	0.51–1.53	0.651

Hazard ratios (HRs) and 95% confidence intervals (95% CI) were estimated using time-dependent Cox regression. Reference categories were: smoking = current smoker; socioeconomic status = low SES; alcohol intake = high; physical activity = inactive; plasma glucose > 7 mmol/L; and falls, osteoarthritis, hypertension, stroke, cardiac arrhythmia, asthma, emphysema, bronchitis, cancer and muscle weakness. Medication reference groups were use of statins, oral glucocorticoids, gonadal hormones, thyroid hormones, and calcium-affecting agents. Selected continuous variables (dietary calcium intake, height, HDL cholesterol, and plasma glucose) were inverted to examine the influence of lower concentrations on fracture risk. Values in bold indicate statistical significance (*p* < 0.05)

BMI, body mass index; HDL, high-density lipoprotein; SES, socioeconomic status; HR, hazard ratio; CI, confidence interval

Exploratory stepwise AIC selection further highlighted age-specific differences in predictor relevance. Among adults aged ≥ 65 years, physical activity (HR 0.74, 95% CI 0.58–0.94, *p* = 0.015) and non-faller status (HR 0.74, 95% CI 0.58–0.94, *p* = 0.015) were selected as significant predictors, alongside hip T-score (HR 0.85, 95% CI 0.74–0.98, *p* = 0.025), spine BMD (HR 0.82, 95% CI 0.76–0.89, *p* < 0.001) and non-use of oral glucocorticoids (HR 0.51, 95% CI 0.31–0.84, *p* = 0.008). In adults aged 20–64 years, stepwise selection identified spine T-score (HR 0.79, 95% CI 0.62–1.00, *p* = 0.048), low HDL (HR 0.61, 95% CI 0.38–1.00, *p* = 0.052), low plasma glucose (HR 0.33, 95% CI 0.15–0.71, *p* = 0.004), asthma (HR 0.44, 95% CI 0.29–0.67, *p* < 0.001), muscle weakness (HR 0.32, 95% CI 0.10–1.03, *p* = 0.055) and non-use of oral glucocorticoids (HR 0.31, 95% CI 0.13–0.72, *p* = 0.007) as the most relevant predictors. These patterns reinforce that fragility-related factors (physical activity, falls and skeletal measures) are more influential in older adults, whereas metabolic, respiratory and neuromuscular factors are more relevant among participants aged 20–64 years (Table 5).

**Table 5:** Predictors selected by stepwise Cox regression using forward selection for protective factors of fractures, stratified by age group

Predictors	20–64 years			Above 65 years		
	HR’s	95%CI	*p*	HR’s	95%CI	*p*
Physically active	–	–	–	0.74	0.58–0.94	**0.015**
Hip T-score	0.79	0.62–1.00	**0.048**	0.85	0.74–0.98	**0.025**
Spine T-score	0.79	0.66–0.95	**0.011**	0.82	0.76–0.89	**< 0.001**
Low serum HDL (mmol/L)	0.61	0.38–1.00	0.052	0.76	0.56–1.02	0.064
Low plasma glucose (< 7mmol/L)	0.33	0.15–0.71	**0.004**	0.67	0.45–1.02	0.063
Non-faller	0.70	0.46–1.08	0.107	0.74	0.58–0.94	**0.015**
No asthma	0.44	0.29–0.67	**< 0.001**	–	–	–
No bronchitis	–	–	–	1.96	0.87–4.42	0.104
No muscle weakness	0.32	0.10–1.03	0.055	–	–	–
Non-use of oral glucocorticoids	0.31	0.13–0.72	**0.007**	0.51	0.31–0.84	**0.008**

Hazard ratios (HRs) and 95% confidence intervals (95% CI) were estimated using time-dependent Cox regression. Reference categories were: smoking = current smoker; socioeconomic status = low SES; alcohol intake = high; physical activity = inactive; plasma glucose > 7 mmol/L; and falls, osteoarthritis, hypertension, stroke, cardiac arrhythmia, asthma, emphysema, bronchitis, cancer and muscle weakness. Medication reference groups were use of statins, oral glucocorticoids, gonadal hormones, thyroid hormones, and calcium-affecting agents. Selected continuous variables (dietary calcium intake, height, HDL cholesterol, and plasma glucose) were inverted to examine the influence of lower concentrations on fracture risk. Values in bold indicate statistical significance (*p* < 0.05)

HDL, high-density lipoprotein; HR, hazard ratio; CI, confidence interval

## Discussion

In this study, predictors associated with reduced fracture hazard were identified, providing valuable insights into potential predictors of major incident fractures during adulthood. Overall, the findings show that males were linked to a reduced hazard of fractures compared with females. The results also indicate that both skeletal and non-skeletal factors contribute meaningfully to fracture hazard. Higher BMD at the hip and spine was associated with a reduced hazard of fracture, reinforcing the central role of bone density in maintaining skeletal resilience. In addition, lifestyle, anthropometric, metabolic and clinical characteristics, including never smoking and smoking cessation, lower serum HDL, lower plasma glucose, not falling, absence of muscle weakness and non-use of oral glucocorticoids, were associated with reduced fracture hazard. Collectively, these results highlight the multifactorial nature of fracture susceptibility and emphasise the importance of integrating bone health, metabolic status and functional capacity when considering fracture prevention across adulthood.

Among these identified predictors, smoking status was strongly associated with fracture hazard in this cohort, with never-smokers and past smokers showing substantially lower hazards compared with current smokers. Previous prospective studies have reported similar patterns, with current smokers experiencing 30–50% higher fracture hazard and accelerated bone loss compared with non-smokers, largely due to impaired bone turnover, reduced BMD, and earlier menopause in women [19]. Although not all studies have observed consistent associations, several have suggested that the relationship between smoking and fracture hazard may be partly mediated by BMD, particularly among women. Variability in findings across the literature often reflects differences in how smoking exposure was measured (e.g., pack-years vs. current smoking) and limited statistical power to detect dose–response effects. A key limitation of many previous studies is their reliance on a single, static measurement, which may not reflect changes over time. In contrast, our analysis incorporates time-updated covariates at two follow-up intervals, allowing us to capture temporal variation and better account for dynamic changes in exposure. However, similar to much of the existing literature, we were unable to assess dose dependency, which remains an important unresolved limitation.

While lower physical activity was observed among participants who fractured in the baseline comparison, physical activity did not remain an independent predictor in the fully adjusted time-dependent model. This null association should be interpreted cautiously. Although physical activity tended to be associated with higher BMD and reduced fracture hazard [20, 21], the measure available in this cohort lacked information on frequency, duration and intensity, limiting its ability to capture meaningful variation in exposure. In addition, physical activity is correlated with several musculoskeletal and metabolic factors included in the multivariable model, raising the possibility of residual confounding or overadjustment. The wide adult age range (20–96 years) may also contribute to heterogeneity in the relationship between physical activity and fracture risk, with effects potentially differing across the life course. Together, these factors likely explain the attenuation of the association in adjusted models and do not contradict established evidence supporting physical activity as beneficial for musculoskeletal health.

Similarly, lower waist circumference was retained in the stepwise Cox regression despite a p-value above the conventional threshold, likely reflecting its overall contribution to the predictive structure of the model. These findings align with a growing body of evidence suggesting that excessive abdominal adiposity may be a more sensitive indicator of fracture risk than BMI alone [22]. While higher body mass has traditionally been associated with a reduced risk of fractures through increased mechanical loading and higher BMD, recent evidence has challenged this obesity paradox [23]. A study reported that, after adjusting for BMI, each one-unit increase in waist circumference is associated with a higher hazard of fracture [24]. Moreover, abdominal obesity has also been linked to site-specific risks, including a 12% increased risk of distal lower limb fractures in women [22]. However, waist circumference is prone to measurement error, as values can vary depending on the protocol used, making it difficult to discern true clinical differences [25].

Regarding biochemical markers, lower HDL cholesterol revealed a significant association with a reduced fracture hazard. While higher HDL concentration is considered protective for cardiovascular health, its role in bone health may be more complicated [26]. Our findings might be explained by experimental and epidemiological studies that suggest HDL cholesterol may influence bone metabolism through modulation of inflammatory pathways and lipid oxidation [27]. A recent study using the ASPREE data revealed similar findings, which show an elevated fracture risk at higher HDL concentrations in a sample of older people [28]. Meanwhile, a question on the complexity of HDL was raised in recent studies, with some evidence showing extremely high HDL levels in association with adverse outcomes such as all-cause mortality, atherosclerotic cardiovascular disease (CVD) mortality, coronary artery disease mortality and stroke [29, 30].

Additionally, metabolic health indicators demonstrated notable and consistent associations with fracture hazard. This finding aligned with previous studies which reported that lower plasma glucose was associated with reduced hazard of fractures, reinforcing growing evidence that dysglycaemia and insulin resistance may compromise bone quality through mechanisms such as altered collagen cross-linking and impaired microarchitecture [31]. Although we did not consider glucose-lowering medications in our model, prior research indicates that thiazolidinediones, metformin and GLP-1 receptor agonists may influence skeletal outcomes and suggests that broader metabolic regulation may contribute to fracture susceptibility [32]. Additionally, this study lacks longitudinal data on HbA1c levels, which would help provide a more stable measure of long-term glycaemic control than a single plasma glucose reading.

Beyond lifestyle factors, anthropometric measures and biochemical markers, higher BMD at the hip and spine demonstrated a robust and clear association with reduced fracture risk. This aligns with extensive evidence showing that greater BMD is strongly associated with lower risk of fractures [33]. Our results are consistent with a meta-analysis which indicates that for every one-standard deviation (1-SD) decrease in BMD, the risk of fracture increases approximately 1.5 to 2.5-fold, depending on the site [34]. It should be noted that although low BMD is a strong predictor of fracture risk, many fractures in our cohort occurred in individuals with BMD in the normal or osteopenic ranges. This highlights a critical limitation of relying solely on BMD in predicting fractures [35].

Comorbid conditions contributed variably to fracture hazard, with the absence of falls showing a strong association with reduced fracture hazard, reaffirming falls as a central mechanism underlying fracture occurrence [36]. Our findings align with well-established literature that even in individuals with high BMD, a single fall event increases the risk of fracture [37]. Furthermore, having no history of asthma was associated with a reduced fracture hazard, though borderline significant but aligned with the literature linking chronic obstructive pulmonary conditions with bone loss, a process that can be mediated by systemic inflammation, reduced physical activity and corticosteroid use [38]. Some large studies report similar findings, primarily attributing this risk to the use of oral corticosteroids [39]. More recent evidence from the GLOW study suggests that the disease state itself, characterised by chronic systemic inflammation, is a strong independent predictor of fracture [40]. Our finding that no history of muscle weakness was associated with reduced fracture hazard is consistent with the findings from the Sarcopenia and Hip Fracture Study, which reported that individuals with normal grip strength and gait speed had approximately a 40% lower risk of fracture compared with those meeting sarcopenia criteria [41].

Medication exposures demonstrated some of the strongest associations observed in the study. Specifically, non-use of oral glucocorticoids showed a strong inverse association with fracture hazard, reflecting the well-established detrimental effects of glucocorticoids on bone turnover, muscle strength and fall risk [42]. Recent evidence further indicates that even low-dose, prolonged glucocorticoid exposure is associated with a higher hazard of fracture compared with non-use, a trend reflected in the lower fracture hazard among non-users of oral glucocorticoids [43].

Age-stratified sensitivity analyses provided additional clinical context to the associations observed in the primary model. In the full cohort, lower hip and spine BMD, low HDL, low plasma glucose, non-faller status, muscle weakness and non-use of oral glucocorticoids were independently associated with reduced fracture hazard. By age group, several predictors showed different patterns of relevance. Among adults aged 20–64 years, spine BMD, low HDL, low plasma glucose, asthma, muscle weakness and non-use of oral glucocorticoids remained significant, reflecting the greater contribution of metabolic, respiratory and neuromuscular factors to fracture risk in mid-life. In contrast, among adults aged ≥ 65 years, male sex, hip and spine BMD, non-faller status and non-use of oral glucocorticoids were the principal predictors, consistent with the established role of skeletal fragility and fall-related mechanisms in older adults [44]. Notably, physical activity, non-significant in the primary model, emerged as a relevant predictor in exploratory stepwise selection among adults aged ≥ 65 years, aligning with evidence that mobility limitation and reduced physical activity contribute to frailty, impaired balance and fall risk in later life [45]. Conversely, predictors such as asthma, low plasma glucose and muscle weakness, which were significant in mid-life, were not independently associated with fracture hazard in older adults, likely due to collinearity with frailty-related variables and multimorbidity. These findings collectively suggest that the predictors of fracture hazard shift across the life course, with metabolic and neuromuscular factors more influential in mid-life, and skeletal fragility, falls and functional status more prominent in older adults.

The study contributes methodologically by integrating repeated measures over long follow-up and applying time-updated covariates with age as the underlying time scale to examine biological, behavioural, socioeconomic, metabolic and clinical factors associated with fracture hazard. This study has several strengths and weaknesses. First, the study used a large, well-characterised, population-based cohort with broad age coverage (20–96 years) and long follow-up, enabling robust estimation of fracture risk across adulthood. Large osteoporosis cohorts such as the Japanese Population-based Osteoporosis Study (JPOS), the Canadian Multicentre Osteoporosis Study (CaMOS), the Osteoporotic Fractures in Men Study (MrOS), the Dubbo Osteoporosis Epidemiology Study and the Perth Longitudinal Study of Ageing Women (PLSAW) have examined a range of demographic, lifestyle and clinical predictors of fracture. While these studies have provided important insights, most relied on a single baseline assessment or a cross-sectional study design and did not incorporate repeated measures of metabolic, inflammatory or body-composition markers over time. In contrast, the availability of repeated measures in the present study enabled the use of time-dependent Cox models, allowing a more accurate representation of dynamic changes in risk factors over the follow-up period. This comprehensive modelling strategy, including multivariable adjustment, assessment of multicollinearity and stepwise selection, enhanced the robustness and interpretability of the findings. The data obtained from the radiological records ensured high-quality, radiologically verified fracture outcomes, reducing misclassification bias. However, some fractures might have been managed outside the region and were, therefore, not captured by our protocol. Further, we identified fractures only at the hip, spine, proximal humerus, distal radius and pelvis; fractures at other sites were not considered.

A key methodological limitation is the handling of death as a competing event. In a cohort with long follow-up and an ageing population, mortality can preclude the occurrence of fracture and therefore influence risk estimates. Because our analysis required time-dependent covariates to capture changes in risk factors over time, competing-risk Fine–Gray models were not feasible. We therefore used a cause-specific Cox approach in which participants were censored at the time of death. While appropriate for identifying predictors of fracture, this approach may affect absolute risk estimates, particularly among older participants with higher mortality. As such, the potential impact of competing risk by death should be considered when interpreting the findings.

Due to the observational nature of the data, we are unable to make inferences about causality, and some relevant risk factors may be unmeasured or not captured within our dataset. The study is geographically restricted to a single region in southeastern Australia, which may limit generalisability to more diverse populations. Potential reporting bias may also arise from self-reported measures, and the absence of information on the frequency or duration of physical activity prevents evaluation of specific activity levels or dose-response effects. A key limitation shared with previous research is the reliance on retrospective self-reported fall history; older adults underreport falls by 20–30%, particularly when no injury occurs, which may underestimate the true protective effect of remaining fall-free [46]. Additionally, while our model accounts for glucose levels, it does not distinguish between the direct effects of hyperglycemia and the indirect effects of exogenous insulin or other glucose-lowering medications, which are known to have independent and sometimes opposing effects on bone turnover [47]. Future studies should explore the mechanisms underlying the potential role of HDL in fracture risk and incorporate larger, more diverse longitudinal cohorts with comprehensive covariate data to validate these findings.

## Conclusion

Fracture hazard in this study was primarily shaped by metabolic and behavioural factors, including bone density, blood lipid and glucose profiles, smoking history and falls. Metabolic indicators, particularly plasma glucose, showed strong associations with fracture hazard, consistent with emerging evidence that dysglycaemia and insulin resistance may adversely influence bone quality. Age-stratified analyses further demonstrated that the pattern of associations differed across the life course. In adults aged 20–64 years, metabolic, respiratory and neuromuscular characteristics, such as low HDL, low plasma glucose, asthma and muscle weakness, showed stronger associations with fracture hazard. In contrast, among adults aged ≥ 65 years, skeletal fragility and fall-related factors predominated, with hip and spine BMD, fall history and physical activity showing the most consistent associations. These findings align with clinical evidence that fragility fractures in older adults arise from the interplay between declining bone strength, impaired balance, reduced mobility and multimorbidity. The unexpected association between lower HDL and reduced fracture hazard should be interpreted cautiously and warrants further investigation to clarify underlying mechanisms. Continued research is needed to better understand the biological pathways linking these determinants and how they interact over time to influence skeletal resilience.

## Data Availability

Data are available upon reasonable request.
